# Double edged effect of gum-resin of ferula assa-foetida on lifespan of neurons

**Published:** 2013-04

**Authors:** Farshad Homayouni Moghadam, Behzad Vakili Zarch, Mohammad Shafiei

**Affiliations:** 1 Neurobiomedical Research Center, Shahid Sadoughi University of Medical Sciences, Yazd, Iran; 2 Department of Physiology, School of Medicine, Shahid Sadoughi University of Medical Sciences, Yazd, Iran

**Keywords:** Asafoetida, Mesenchymal stem cells, Neuroprotective, Neurotoxic

## Abstract

***Objective(s):*** Based on knowledge from traditional herbal medicine, Ferula assa-foetida (asafoetida) has several therapeutic applications but there is less knowledge about its effect on neurons.

***Materials and Methods:*** In order to evaluate neuronal differentiation, neuronal like cells were stained against neuronal specific markers β-Tubulin III and MAP2. After establishment of neuronal differentiation in cultured cells, aqueous extract of gum-resin of asafoetida were applied on culture medium of neurons with different concentrations then survival rate of neurons were evaluated by cell counting and methyl tetrazolium bromide (MTT) tests.

***Results:*** The results showed that asafoetida gum resin particularly with 0.01 and 1 µg/ml concentrations could improve survival rate of neurons, while10 µgr/ml treated group was toxic.

***Conclusion:*** Results of this study indicated that gum resin of asafoetida in low doses has neuroprotective effect on neurons and improves survival rate of them, however in higher concentrations it is toxic for neurons.

## Introduction

Ferula assa-foetida (asafoetida) is a plant which is growing in Middle East countries, and its Gum resin has some therapeutic effects according to Iranian, Indian and Chinese traditional medicines (1). About indicating effects of asafoetida on nervous system, there are some evidence from Ayurveda, representing the application of asafoetida in epilepsy, anxiety and emotional disorders (1). American people use it as a nervous stimulant, but in Nepal, it is used as a sedative (1). As a result, it might have both calming and stimulatory effects together.

In cellular level, its effects on neuronal cells were not studied yet. But, another double-edged effect could be predictable according to its gum resin compositions; which contains both surviving and toxic factors (1). The neuroprotective effects could be attributed to ferulic acid that is one of its active compositions (1), while other active compounds that are exist in many plants could be also play neuroprotective role (2).

Some kind of sulfur compounds like disulfides, as well as symmetric tri- and tetra sulfides have been isolated from Asafoetida (3). Effect of sulfur compounds on neurons could be conflicting. It was shown in some experiments that sulfur compounds could exert protecting effects on neurons (4)**, **but it is predictable that in presence of extreme amounts of sulfurous compounds (greater than the capacity of the body sulphite oxidase system) excess sulfur could enter animal’s bloodstream as sulfites, which are very toxic for neurons (5).

In present study we investigated the effect of different concentrations of asafoetida resin, solved in culture medium, on the survival rate of neurons. Neurons were produced from rat mesenchymal stem cells (MSCs) by using chemical induction methods (6). The adult bone marrow stem cells or MSCs have potentiality for self-renewal and proliferation (6). Under specified conditions, they could differentiate to multiple cell lineages, such as osteoblasts, adipocytes, chondrocytes, myoblasts, hepatocytes and neurons (6-9). There are some induction protocols to generate neurons from these cells, and by using some of them there would be a high neuronal differentiation rate (6,7). These neurons were named neuron-like cells primarily, but complementary studies demonstrated that these neuron-like cells could exhibit several electrophysiological key properties regarding to neurons including expression of neuronal markers and firing of action potentials (7). These cells are easy producible and it makes them a good source for in-vitro drug screening studies.

## Materials and Methods

*Cell culture*

All media were purchased from Gibco (Invitrogen), unless otherwise specified. Bone marrow was obtained from tibia and femoral bones of Adult Wistar rats (weighting 250-300g) by aspiration, and then suspended in 10 ml of Dulbecco’s modified Eagle’s medium (DMEM). Cells were centrifuged, and 10^8 ^cells were seeded on 10 cm tissue culture plates. After 24 hr incubation in DMEM+10% fetal bovine serum (FBS), just mesenchymal stem cells were the cells which attached to culture plates and non-adherent cells were removed by medium change. Cells were cultured for 7-10 days to became confluent, and then they were re-suspended with 0.25% trypsin in 1mM EDTA and sub-cultured for several times. MSCs with passage 4-6 were used for neuronal induction (6).

For neuronal induction MSCs were seeded at 1×10^5^ cells per well in 6-well plates and were incubated in 5% CO_2_ and 37°C temperature with 95% humidity in DMEM+10%FBS. Next day cells were cultured in pre-induction medium containing DMEM supplemented with 0.01% β-mercaptoethanol (2ME, Sigma) and 20% FBS for 24 hr, neuronal induction continued by 5 hours incubation in induction serum free medium consisted of DMEM plus 2% dimethylsulfoxide (DMSO, Sigma) and 0.1% 2ME (6). Medium changed after 5 hours with Neurobasal medium (NB) supplemented with 2mM glutamine, 0.1mM non-essential amino acids , 1% N2 supplement, and 1% FBS. To evaluate the effect of asafoetida on neurons, 1mg/ml stock solution of asafoetida was prepared by solving its resin-gum in NB medium and filtered by using 0.2µm filter. Then above solvent added to the culture medium of neurons with different ratios to acquire final concentrations of: 0, 0.001, 0.01, 0.1, 1 and 10 µg/ml. Cells were cultured for 48 hr in mediums containing asafoetida, above experiments repeated three times for each group of asafoetida treatments.

*Immunocytochemical staining*

Cells were stained against neuronal specific markers β-Tubulin III, MAP2, and cells nuclei counterstained with Propidium Iodide (PI). Immunofluorescent staining were performed after ﬁxation of cells with cold 4% paraformaldehyde (Sigma; P6148) for 10 min then cells were rinsed three times with PBS–tween-20 (0.05%) followed by 30 min incubation with 5% goat normal serum +0.3% bovine serum albumin+0.25% Triton X-100 in Tris-buffered saline (TBS). Staining continued by overnight incubation at 4°C with primary antibodies; microtubule associated protein 2 (Map2, Sigma M2320, 1:250) and β-Tubulin III (Sigma T8660, 1:200) diluted in BSA (0.1%)-PBS-tween20 (0.05%), then, after three times rinsing with PBS–tween-20 (0.05%), cells were incubated with FITC-conjugated secondary antibody goat anti-mouse (Chemicon, AP124F, 1:100), diluted in BSA (0.1%)-PBS-tween20(0.05%), for 30 min at room temperature. Nuclei of cells were counterstained by 5 min incubation with Propidium Iodide (PI, 0.2 mg/ml, Sigma; P4170) followed by 3 times rinsing with PBS-tween-20. After staining 20 pictures captured from 20 different sites of each culture well with 20× magnifications and number of cells stained against Map2 and PI were counted in each image. 

*Measurement of cell viability*

Cell viability measured with the 3-(4,5-dimethyl-2thiazolyl)-2,5-diphenyl-2H-tetrazolium bromide (MTT, Sigma) assay which is indicating the cellular mitochondrial dehydrogenase activity in living cells (10). For this purpose every sample test repeated 6 times for each treatment group. Two days after initiation of asafoetida treatment, cells in each well of 6-well culture dishes were exposed to 1ml MTT (5mg/ml) in phenol red free medium and incubated for 2 hr. Culture mediums were removed and replaced with 1 ml of DMSO. Absorbance of each well was measured at 570nm by spectrophotometer and results expressed as percentage of control.

*Statistical analysis*

The data collected from cell counts were presented as: number of cells stained against Map2, and ratio of Map2/PI stained cells for each image, following ratio considered as a neuronal differentiation rate. The data were analyzed using paired student t-test. All the data expressed as mean ± standard error of mean (SEM), and *P<*0.05 considered as significant.

## Results

*Effect of DMSO and 2ME on neuronal differentiation*

Five hours after exposure to 2%DMSO and 0.1% 2ME there was a great change in morphology of MSCs and they acquired neuronal phenotype. These cells also expressed neuronal specific markers Map2 and β-Tubulin III. Five hours after initiation of induction one sample group of cells stained against β-Tubulin III and MAP2 and nuclei stained by PI and the ratios of stained cells against non-stained cells were 92.35% ±9.61% for β-Tubulin III and 88.49%±7.83% for MAP2.

*Effect of asafoetida on survival rate of neurons*

**Figure 1:**
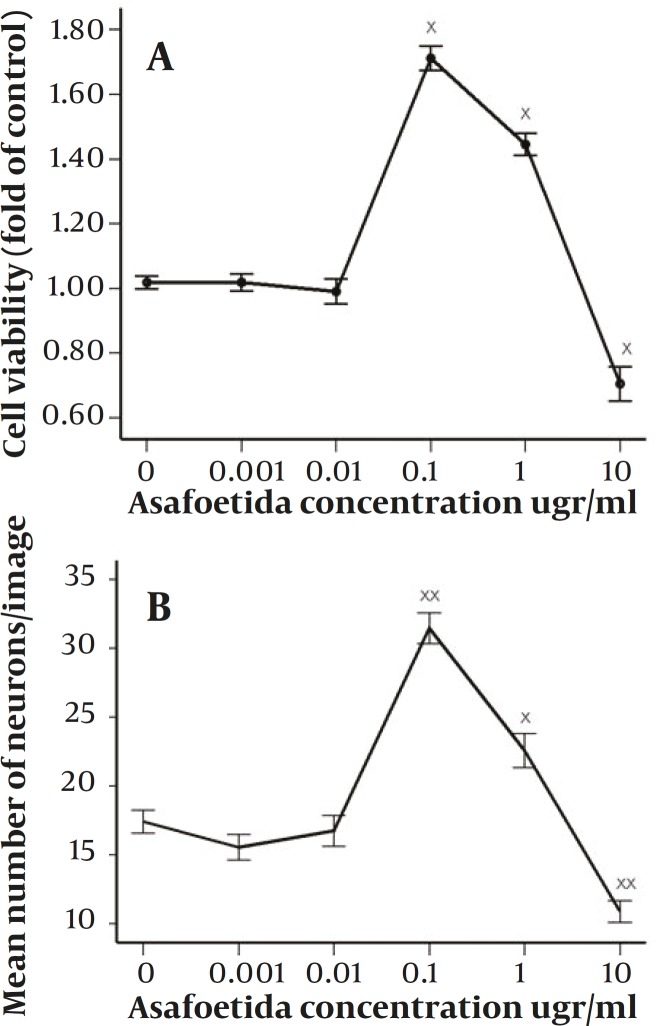
A: effect of 48h treatment with 0–10µg/ml of Asafoetida on survival rate of neurons derived from rat MSCs (×<0.05). The relative number of cells per well was determined by MTT assay. B: mean number of neurons stained against Map2 in images after 48h treatment with 0–10 µg/ml of Asafoetida (×<0.001 & ××<0.0001). The data presented as the mean ± SEM

**Figure 2:**
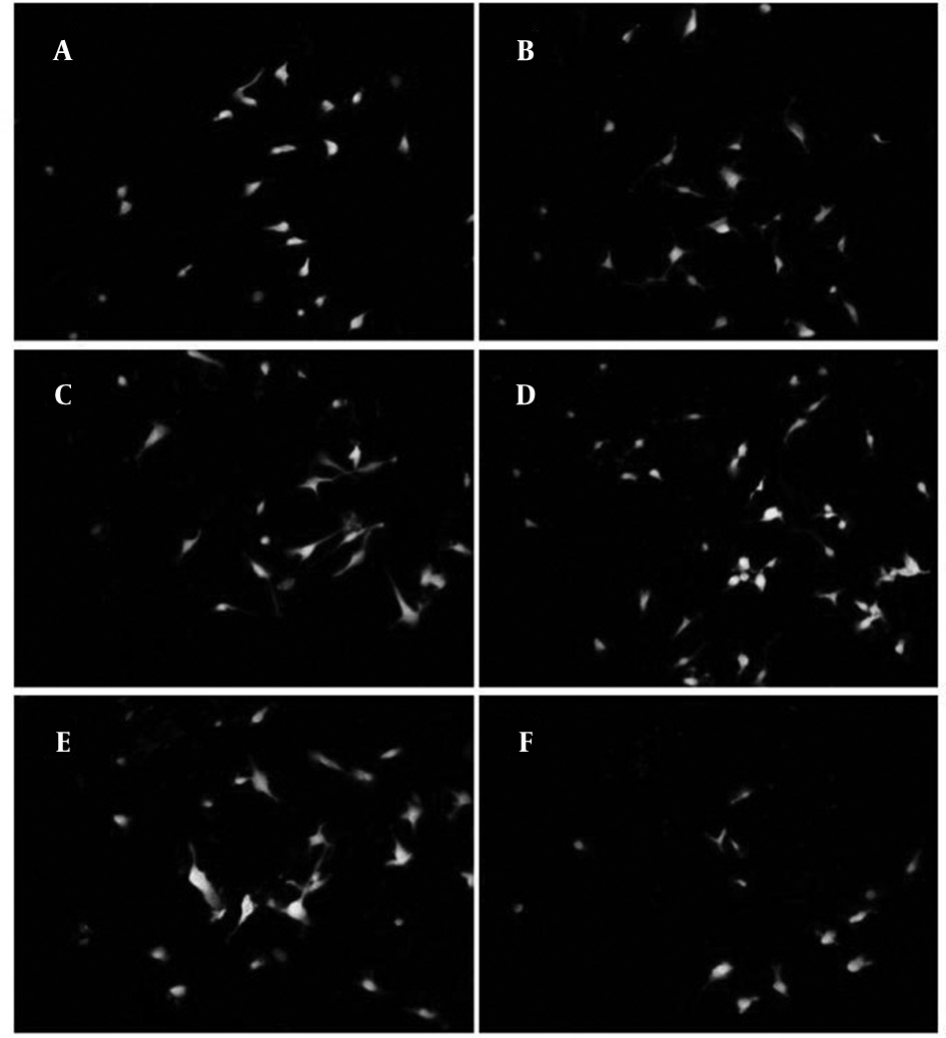
Cells treated with different concentrations of asafoetida and stained against Map2 (green) and PI (red). Treatment groups were: A: 0, B: 0.001, C: 0.01, D: 0.1, E: 1 and F: 10µgr/ml of asafoetida. There were higher numbers of neurons in D&E compared to other groups. Magnification 20X

According to previous studies, forty eight hours after induction there was an apparent cell death in all treated and not treated groups (12). Based on MTT assay (Figure 1A), in some groups especially in 0.1 µg/ml treated group this cell death was lower and it was significantly different compared with non-treated group (*P<*0.001), in 1 µg/ml treated group there was also higher rate of survival and was statistically significant compared with control (*P<*0.001). However, the survival rate in 1 µg/ml treated group was not as high as 0.1 µg/ml treated group. On the other hand, in 10 µg/ml treated group there was higher rate of apoptosis compared with control (*P<*0.004). While, in 0.01 & 0.001 µg/ml treated groups there were not statistically significant differences compared with control. According to the results of neuronal cell count in different groups, the ratios of neurons against other cells were not changed significantly between all groups. But there were apparent changes between groups in total number of neurons counted in each image (Figure 1B). Asafoetida with 0.1 µg/ml concentration in culture media strongly prevents this cell death as shown in figure 2. Mean number of MAP2 stained neurons per each image were 31.46±8.65 and 22.57±9.59 in 0.1 µg/ml and 1 µg/ml treated groups respectively and they were statistically different in comparison with control group 17.41±6.49 (*P*≤0.0001 and *P*≤0.001). In other groups of treatment, 0.01 and 0.001 µg/ml, number of neurons was near to control 16.75±8.59 and 15.54±7.17 respectively. While in 10 µg/ml treated group there was lower rate of neurons, 10.89±5.99 compared with control (*P<*0.0001).

## Discussion

Above data indicates that asafoetida; dose dependently has both neuroprotective and neurotoxic effects on neuronal like cells. Resin-gum of asafoetida, solved in the culture medium of neurons, exerts protective effects in low doses between 0.1-1 µg/ml, while it was neurodegenerative in doses above 1 µg/ml. Neuroprotective effects could be attributed to existence of different kinds of neutraceuticals such as: 1) flavonoids, 2) phenolic acids, and 3) polysulﬁde compounds (1). Antioxidative effects of the compounds belonging to above categories were established in some studies and several mechanisms were explained for them. Certain flavonoids could inhibit NO production via reduction of inducible nitric oxide synthase (iNOS) expression (11). Asafoetida is full of sulfurous compounds and surviving effect of polysulfide compounds on neurons derived from mouse embryo was reported previously (2). Sulfur-containing neutraceuticals, having neuroprotective effects, may exert some direct antioxidative effects, their principal mode of neuroprotection is through activation of endogenous antioxidant systems, including gene targets of the Nrf2/ARE (Nrf2-antioxidant response element) transcription factor pathway (12). Additionally, some other components like sesquiterepene coumarins (1), sodium ferulate (13), and ferulic acid are also neuroprotectice. Ferulic acid could improve survival rate of neurons through inhibiting ICAM-1 mRNA expression (14). The sesquiterepene coumarins such as fukanefuromarin B, E, F and G could suppress No production (15). Therefore, inhibition of NO production by flavonoids and sesquiterepenes strongly removes NO from microenvironment and increases endurance of neurons in culture. 

On the other hand, for neuronal differentiation of mesenchymal stem cells we cultured them in the serum free medium supplemented with high concentrations of 2ME and DMSO. This method of neuronal induction could cause cell stress and apoptosis (6). Neutraceuticals could improve survival rate of these neurons by modulating signaling cascades such as pro-survival Bcl-2, MEK/ERK and PI3K/AKT which finally could reduce inflammatory and apoptotic signals (2). 

Despite above evidences, our study established that asafoetida could be neurotoxic in a moderate to high doses. By increasing the dose of asafoetida, effect of unwanted components will overcome and could cause neuronal death. These unwanted chemicals could be some harmful derivatives of above explained compositions like sulfites (5) and galbanic acid which are very toxic elements (1). But further studies need to be done to distinguish which factors is exactly neurotoxin. 
